# Differences in Acid Stress Response of *Lacticaseibacillus paracasei* Zhang Cultured from Solid-State Fermentation and Liquid-State Fermentation

**DOI:** 10.3390/microorganisms9091951

**Published:** 2021-09-14

**Authors:** Pengyu Wu, Qiuyan Zhu, Rui Yang, Yuxia Mei, Zhenmin Chen, Yunxiang Liang

**Affiliations:** State Key Laboratory of Agricultural Microbiology, College of Life Science and Technology, Huazhong Agricultural University, Wuhan 430070, China; 18771068351@163.com (P.W.); 15575550652@163.com (Q.Z.); kingsray@163.com (R.Y.); mei@mail.hzau.edu.cn (Y.M.); zmchen@mail.hzau.edu.cn (Z.C.)

**Keywords:** *Lacticaseibacillus paracasei* Zhang, acid stress tolerance, solid-state fermentation, liquid-state fermentation, transcriptomics

## Abstract

Liquid-state fermentation (LSF) and solid-state fermentation (SSF) are two forms of industrial production of lactic acid bacteria (LAB). The choice of two fermentations for LAB production has drawn wide concern. In this study, the tolerance of bacteria produced by the two fermentation methods to acid stress was compared, and the reasons for the tolerance differences were analyzed at the physiological and transcriptional levels. The survival rate of the bacterial agent obtained from solid-state fermentation was significantly higher than that of bacteria obtained from liquid-state fermentation after spray drying and cold air drying. However, the tolerance of bacterial cells obtained from liquid-state fermentation to acid stress was significantly higher than that from solid-state fermentation. The analysis at physiological level indicated that under acid stress, cells from liquid-state fermentation displayed a more solid and complete membrane structure, higher cell membrane saturated fatty acid, more stable intracellular pH, and more stable activity of ATPase and glutathione reductase, compared with cells from solid-state fermentation, and these physiological differences led to better tolerance to acid stress. In addition, transcriptomic analysis showed that in the cells cultured from liquid-state fermentation, the genes related to glycolysis, inositol phosphate metabolism, and carbohydrate transport were down-regulated, whereas the genes related to fatty acid synthesis and glutamate metabolism were upregulated, compared with those in cells from solid-state fermentation. In addition, some genes related to acid stress response such as *cspA*, *rimP*, *rbfA*, *mazF*, and *nagB* were up-regulated. These findings provide a new perspective for the study of acid stress tolerance of *L. paracasei* Zhang and offer a reference for the selection of fermentation methods of LAB production.

## 1. Introduction

In daily life, people always come into contact with lactic acid bacteria (LAB) and corresponding products. Due to their safety [1,2], they are widely used as probiotic products to improve the balance of human intestinal microbiota and promote human health [3,4,5,6,7,8]. With the ban on antibiotics in feed, LAB are also used as feed additives to substitute for antibiotics and to promote the health of farmed animals [9,10,11,12,13]. Sufficient viable bacteria are the prerequisite for LAB to function as probiotics, and in the process of production, processing, and food consumption, a large number of living LAB will die. Therefore, how to obtain high concentration of LAB by fermentation and maintain their high survival rate in processing and application has become the interesting topic for researchers and commercial companies. At present, the production of LAB is mainly divided into liquid-state fermentation (LSF) and solid-state fermentation (SSF). Liquid-state fermentation is widely used in food industry, medicine and health industry because of its high fermentation density and low contamination by miscellaneous bacteria [14,15]. SSF can reduce the metabolite decomposition inhibition and substrate fermentation inhibition, and it has multiple advantages, such as high enzyme production, low energy consumption, high product stability, low production cost, a wide range of raw material sources, and ecological friendliness [16,17,18,19,20]; therefore, SSF is widely used. The growth of LAB is accompanied by the production of organic acids, which seriously affects their own growth and decreases their number [21]. In the final application stage, LAB are also faced with the acidic environment of the human or animal stomach, thus resulting in a significantly decreased number of viable bacteria and a weakened performance as probiotics [22]. Therefore, improving the acid stress tolerance of LAB is of great significance for their industrial production and application.

With the extensive application of genome, transcriptome, and metabolomic techniques in recent years, the understanding of the acid tolerance mechanism of LAB has been deepened [23,24]. Existing studies have revealed that LAB can resist acid stress by changing the types and contents of fatty acids in cell membranes to reduce the cell damage caused by the external environment [25]. Acid preadaptation of *Lactobacillus plantarum* ZDY2013 (recently reassigned as *Lactiplantibacillus plantarum* ZDY2013) has been reported to significantly increase the proportion of saturated fatty acids and cyclopropane [26] and to enhance the function of proton pump. When the external pH value is low, the expression of H^+^-ATPase gene is up-regulated, and the activity of H^+^-ATPase enzyme is increased, but when H^+^-ATPase is absent, the mutant cannot grow normally in acidic conditions [27]. LAB can improve the pH value of the surrounding environment of cells through arginine deaminase pathway or glutamate decarboxylase system so as to improve the cell living environment [28]. In addition, related mechanisms include protection and repair of nucleic acids and proteins [24,29,30], two-component system regulation, cell density regulation, rigorous response (ppGpp), quorum-sensing, and changes in acid stress-related cell metabolic pathways [29,31,32]. However, most existing studies focus on bacterial stress response after they experience a series of acid stress stimuli, and there is still a lack of studies on how growth conditions before the introduction of acid stress affect acid stress response, and on internal mechanisms of LAB produced through different culture methods in response to environmental stress. However, these absent studies have an important application value for the selection of different culture methods and the production of probiotics and feed products.

In this study, the survival rate of the LAB obtained by solid and liquid fermentation methods and their tolerance difference under acid stress were investigated. The results indicated that bacterial cells from liquid-state fermentation had better tolerance to acid stress. Then, the cell integrity, membrane fatty acid composition, intracellular microenvironment, and important metabolic enzyme activities in the cells from two culture methods were compared before and after acid stress, and the physiological responses of these cells under acid stress were examined. Furthermore, transcriptome technology was used to compare the gene expression differences between the cells produced by two culture methods, and to analyze cells’ metabolic networks. Our findings provide reference for the study of resistance mechanism of *L. paracasei* Zhang and the application of solid and liquid fermentation methods.

## 2. Materials and Methods

### 2.1. Bacterial Strain, Media, and Growth Conditions

*L. paracasei* Zhang was cultured in MRS (de Man–Rogosa–Sharpe) medium containing 10.0 g/L of peptone, 8.0 g/L of beef extract powder, 4.0 g/L of yeast extract powder, 20.0 g/L of glucose, 0.2 g/L of MgSO_4_·7H_2_O, 5.0 g/L of sodium acetate, 2.0 g/L of sodium citrate, 2.0 g/L of K_2_HPO_4_, 0.15 g/L of MnSO_4_, and 1.0 mL of Tween-80. The agar concentration in culture plates was 15 g/L.

Solid-state fermentation (SSF) medium contained 15.0 g soybean meal, 5.0 g wheat bran, and 12.0 mL deionized water. Sixty gram soybean meal, 20.0 g wheat bran, and 800.0 mL deionized water were mixed. The mixture was heated, and boiled for 10 min, filtered with two layers of gauze to obtain 300 mL solution, from which 100 mL was taken as liquid-state fermentation (LSF) medium. *L. paracasei* Zhang from a −80 °C glycerol stock was streaked on MRS agar plate and cultured for 24 h at 37 °C. A single colony of *L. paracasei* Zhang was cultured in 5.0 mL MRS medium overnight at 37 °C for further experiments.

### 2.2. Preparation of L. paracasei Zhang Bacterial Suspensions after Solid-State and Liquid-State Fermentatio, Cell Crushing Supernatant, and Cell Debris

The overnight cultured *L. paracasei* Zhang was inoculated into 32.0 g fresh SSF medium and 100.0 mL LSF medium with 1.0% inoculum size and cultured for 14 h at 37 °C.

For SSF bacterial suspension preparation, firstly, bacteria were collected from the SSF medium. Then, 80.0 mL sterilized saline water (0.85%) was added into the SSF system and shaken at 200× *g* for 5 min at 37 °C. Afterwards, the mixture was filtered by 2-layer gauze, and the filtrate was centrifuged at 5000× *g* for 5 min. The obtained precipitate was washed twice and finally resuspended with sterilized saline water (0.85%) to obtain the SSF bacterial suspension. For LSF bacterial suspension preparation, firstly, bacteria were collected from LSF medium by aspirating the upper layer of culture carefully, then the obtained culture was filtered by 2-layer gauze. The filtrate was centrifuged at 5000× *g* for 5 min. The resultant precipitate was washed twice and finally resuspended with sterilized 0.85% saline water to obtain the LSF bacterial suspension.

The bacterial suspension was centrifuged at 5000× *g* for 5 min, and the resultant precipitate was washed twice with ultra-pure water and resuspended with ultra-pure water. The suspension was put into 5 mL centrifuge tubes with each tube filled with 3 mL. The suspension in centrifuge tubes was subjected to an ice bath, crushed with an ultrasonic crushing instrument for 20 min (SONICS & MATERIALS, INC.), and centrifuged at 12,000 r/min to obtain crushed cells. The supernatant or the cell fragments were extracted for subsequent use.

### 2.3. Bacterial Agent Preparation by Cold-Air Drying and Spray Drying

The concentration of bacterial suspensions was determined by spread plate count method. The 80 g sterilized wheat bran was added into the pre-prepared liquid-state fermented bacterial suspensions (100 mL) for cold-air drying. In contrast, the solid-state fermented bacterial suspensions were directly cold-air dried. Both SSF and LSF bacterial suspensions were dried for 6 h at 30 °C and 10% humidity with wind gear 8–9 on air drying machine YCFZD-2A (Ouyi Electric Appliance co. LTD, Hangzhou, China). The resultant bacterial agents were stirred once every hour to prevent hardening. Finally, weight (*m*) and biomass (*b*) of bacterial agents were determined, and the bacterial survival rate (*S*) after bacterial agent preparation was calculated according to the following formula (1).

(1) $$\mathrm{Survival}\;\mathrm{rate}\;\left(\%\right)\;S=\frac{m\times b}{C\times V}\times 100\%$$

where *C* is concentration of the bacterial suspensions (CFU/mL); *V* is the volume of the bacterial suspensions (mL); *m* is weight of the bacterial agents after cold-air drying (g); and *b* is biomass of bacterial agents after cold-air drying (CFU/g).

For spray drying, the bacterial suspension was centrifuged at 5000× *g* for 5 min and resuspended with sterilized water. Then, 20 g skimmed milk powder was added into the pre-prepared LSF and SSF bacterial suspensions (100 mL). Concentrations of these bacterial skimmed milk suspensions were determined by spread plate count method. After being mixed uniformly, the suspensions were dried at a constant air inlet temperature of 140 °C and air outlet temperature of 60 °C on spray dryer YC-015 (Yacheng Instrument & Equipment Co., Ltd., Shanghai, China) to obtain the bacterial agent. Finally, biomass (*b*) of the bacterial agents was determined and the survival rate (*S*) after spray drying was calculated using formula (2).

(2) $$\mathrm{Survival}\;\mathrm{rate}\;\left(\%\right)S=\frac{b\times m}{B\times V}\times 100\%$$

where *b* is the biomass of the bacterial agents after spray-drying (CFU/g); *m* is the total weight of the spray-dried powder (g); *B* is biomass of bacterial skimmed milk suspension (CFU/mL); and *V* is total volume of the bacterial skimmed milk suspension used for spray drying (mL).

### 2.4. Tolerance to Acid Stress of L. paracasei Zhang Produced from Solid-State and Liquid-State Fermentations

The optical density at 600 nm (OD_600nm__)_ of bacterial suspensions was adjusted to 1.50, and the initial bacterial number was calculated by spread plate count method. In the acid gradient experiment, the bacterial suspensions were centrifuged at 5000× *g* for 5 min, resuspended with sterilized 0.85% saline water at pH 1, 2, 3, 4, 5, respectively, and incubated at 30 °C for 10 min. The bacterial number was counted by spread plate method. The bacterial survival rate was calculated as follows.

Bacterial survival rate (%) = (initial bacterial number − bacterial number after stress treatment)/initial bacterial number × 100%.

### 2.5. Morphology Observation with Transmission Electron Microscope

The sample preparation processes were as follows. The bacterial cultures in the exponential growth phase were placed in a 2 mL centrifuge tube, and the bacteria were collected by centrifugation at 5000 r/min for 3 min. The collected bacteria were incubated with 2.5% glutaraldehyde solution for 4 h. Afterwards, the solution was suspended to make the cells settle down at the bottom of the centrifuge tube. The centrifuge tube was placed in a refrigerator at 4 °C for 1 week. Taken out of the refrigerator, the cells were treated with 1% osmium tetroxide, fixed in 0.1 M sodium cacodylate buffer for 2 h, and then dehydrated with a graded acetone series (50, 70, 80, 90, and 100%). These dehydrated cells were permeated in the mixture of gradient resin and acetone for 4 days. The permeated samples were embedded, polymerized, cut into ultrathin sections, and stained with uranium 2% acetate solution for 30 min. After sample treatment, cell morphology was observed under transmission electron microscope (HITACHI H-7650).

### 2.6. GC-MS Analysis of CMFA Composition

The cell membrane debris was freeze-dried to prepare fatty acid methyl ester. The 100 mg of cell membrane debris was put into a clean EP tube, and 4 mL (1:1, vol/vol) chloroform methanol solution and 2 mL 0.88% NaCl solution were successively added into EP tube (marked as No. 1), and they were mixed by vortexing for 30 s. The mixture was centrifuged at room temperature at 3500 r/min for 15 min, and stood for 10 min. The lower layer liquid was pipetted and transferred to another clean test tube (marked as No. 2). The 2 mL of methylene chloride was added into the test tube (No. 1), vortexed for 30 s, and centrifuged at 3500 r/min for 15 min. Subsequently, the lower layer liquid from second time centrifugation was transferred into to the same test tube (No. 2) containing liquid from the first-time centrifugation. The lower layer liquid from the two centrifugations was mixed and dried under a flow of nitrogen. After nitrogen drying, 2 mL of methylation reagent (H_2_SO_4_+MeOH) was added into the mixture, and vortexed for 30 s and incubated in water bath at 80 for 2 h. The mouth of the bottle containing the mixture was covered with a low temperature gauze during the water bath to prevent gas from escaping. After the water bath incubation, 2 mL of n-hexane and 1 mL of water were added to the solution, and vortexed for 30 s. The obtained mixture solution was centrifuged for 5 min at 2000 r/min. The up-layer liquid was pipetted and transferred to another clean bottle, 1 mL of ultrapure water was added into the bottle, and vortexed for 30 s, centrifuged at 2000 r/min for 5 min. Subsequently, the resultant supernatant was dried under a flow of nitrogen. The 200 μL of isooctane was added into the dried supernatant, vortexed for 30 s, and then stood for 5 min. The solution was then transferred to the sample bottle for test. GC-MS analysis of samples was completed by Meji Biotechnology Co., Ltd. (Shanghai, China).

### 2.7. Measurement of Intracellular pH(pH_i_), ATP Concentration, Activity of ATPase and Glutathione Reductase

The intracellular pH (pH_i_) in bacterial suspensions was measured by the fluorescence method developed by Guan et al. [33] using 2′,7′-Bis(2-carboxyethyl)-5(6)-carboxyfluorescein tetrakis (acetoxymethyl) ester as the fluorescent probe (MedChemExpress LLC).

After cell crushing, ATP concentration and were determined using an ATP assay kit (Beyotime, Shanghai, China) according to the manufacturers’ instruction. The ATP concentration was expressed as micromole per milligram protein (μM/mg protein).

H^+^-ATPase can decompose ATP to produce ADP and inorganic phosphor. H^+^-ATPase activity was determined with the H^+^-ATPase assay kit (NJJCBIO, China), and the content of inorganic phosphorus was measured at OD_660nm_, according to the manufacturers’ instructions. The results were expressed as U/mg protein. One unit of ATPase referred to the amount of ATPase that produced 1 μM inorganic phosphorus from 1 mg tissue protein per hour at 37 °C by decomposing ATP.

Glutathione reductase (GR) activity was determined by monitoring glutathione-dependent oxidation of NADPH at OD_340nm_ using the glutathione reductase assay kit (Beyotime, China) according to the manufacturers’ instructions. NADPH oxidation corrections were conducted in the absence of GSSG, and the results were expressed as U/mg protein. One unit of GR represented the amount of GR that oxidized 1 nM NADPH per minute at 2 °C.

Protein content was determined using a BCA protein assay kit (Beyotime, China) with bovine serum albumin as standard.

### 2.8. RNA Extraction, RNA-Seq, and Transcriptomic Data Processing

RNA extraction, RNA-seq, and transcriptomic data processing were accomplished by BGI Technology Services Co., LTD (Shenzhen, China). Three biological replicates for each sample were measured.

Total RNA was extracted using TRIzol reagent (Invitrogen, Waltham, MA, USA) and purified with the Rio-Zero rRNA Removal Kit (Illumina, San Diego, CA, USA) following the manufacturers’ instructions. The genomic DNAs were digested by DNase I (Fermentas, Waltham, MA, USA). Degradation and contamination of the as-prepared RNA were monitored on 1.5% agarose gels. RNA concentration was detected with Qubit^®^ RNA Assay Kit in Qubit^®^ 3.0 Flurometer (Life Technologies, Carlsbad, CA, USA). RNA integrity was assessed with the RNA Nano 6000 Assay Kit in Agilent 2100 Bioanalyzer system (Agilent Technologies, Santa Clara, CA, USA) (RIN > 9.0). The RNA samples were added into fragmentation buffer for thermal fragmentation into 130–160 nt. Then, the obtained fragments were reverse transcripted into cDNA with random primers using First Strand Mix, and the second-strand cDNA was generated by Second Strand Mix. The resultant double-strand cDNA was purified, end repaired, amplified, ligated to sequencing adapters, and sequenced on the BGISEQ500 platform (BGI, Shenzhen, China). The sequencing data were filtered using SOAPnuke v1.5.2 and aligned using Bowtie2 [34,35]. The gene expression level analysis and differential expression (DE) analysis were conducted, as described previously [36,37], using *L. casei* Zhang (Firmicutes) (GenBank Accession No. NC_014334.2 and NC_011352.1) as reference genome. *p*-value < 0.001 and fold change >2.0 were standards to define differential expression.

RNA-seq data were deposited in Sequence Read Archive under the accession numbers of SRR12378022 (SSF-1), SRR12378030 (SSF-2), SRR12378029 (SSF-3), SRR12378028 (LSF-1), SRR12378027 (LSF-2), and SRR12378026 (LSF-3).

### 2.9. Statistical Analysis

All presented data were the average of at least three biological replicates. Statistical analysis was carried out using SPSS 20 software. The statistically significant difference was compared by one-way nested analysis of variance (ANOVA), followed by least significant difference test (LSD) for mean comparison. *p*-value < 0.05 was considered as statistically significant.

## 3. Results and Discussions

### 3.1. Differences of Fermentation Biomass, Survival Rate of Bacterial Agent, and Acid Stress Tolerance

Solid state fermentation (SSF) and liquid state fermentation (LSF) methods have their advantages and disadvantages in the production of LAB-related products, and both methods were widely applied. Substrates such as soybean meal, corn, and bran accounted for the majority of global feed consumption [38,39]. Therefore, we used solid and liquid media prepared from bran and soybean meal as substrate to culture *Lacticaseibacillus paracasei* Zhang, then prepared the powder by cold air drying and spray drying, then we detected the survival rate of powder bacterial agent prepared by the two methods (cold air drying and spray drying). The results showed (Figure S1) that the survival rate of the powder bacterial agent prepared by SSF and cold air drying (32.93%) was significantly higher than that prepared by LSF and cold air drying (22.13%). Similarly, the survival rate of the powder bacterial agent prepared by SSF and spray drying (0.21%) was significantly higher than that prepared by LSF and spray drying (0.15%). These results indicated that cold-air drying preparation method was more conducive to the survival rate of LAB than spray drying preparation method with the survival rate increased by two orders of magnitude.

**Figure 1:**
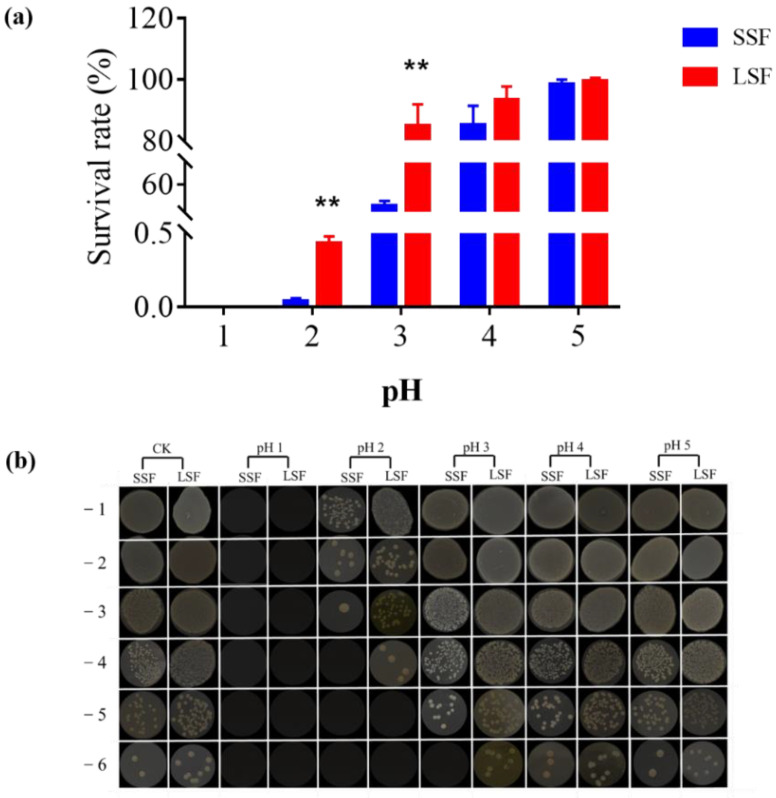
Differences in acid tolerance of *L. paracasei* Zhang cultured from solid-state fermentation and liquid-state fermentation. (**a**) Survival rate of solid-state and liquid-state fermented cells under different pH values; (**b**) Plate diagram of solid-state and liquid-state fermented cells under different pH values. Three biological and three technical replicates were measured. ** statistically significant differences (*p* < 0.01).

Whether it is consumed by human or by cultured animal, the bacteria agent would be digested by the stomach. Acid stress caused by gastric acid poses the biggest challenge for LAB to survive and function as probiotics. Tolerance to gastric acid has become an important indicator to determine whether a strain is a good probiotics strain or not. Therefore, we compared the acid stress tolerance of *L. paracasei* Zhang produced from two fermentation methods. The results showed that (Figure 1), after 10-min pH 5 acid stress treatment, the survival rate of solid-state and liquid-state fermented cells were not basically affected (98.30% and 99.39%, respectively), indicating that the cells had good tolerance to the pH 5. With the increasing acidity, survival rates began to differ. After 10-min pH 4 and pH 3 acid stress, the survival rates of the cells from liquid-state fermentation were 93.34% and 84.99%, whereas the survival rates of the cells from solid-state fermentation were decreased to 85.04% and 39.89%, suggesting that liquid-state fermentation contributed to enhancing the tolerance to acid stress of the cells, compared with solid-state fermentation. After 10-min pH 2 acid stress treatment, the survival rate of the cells from the two fermentation methods showed a sharp decline, and the survival rate of the cells from LSF was only 0.43%, but it was still significantly higher than that of the cells from SSF (0.04%). No viable bacteria were detected after 10-min pH 1 acid stress treatment, indicating bacteria from both fermentations were intolerant to pH 1.

Solid state fermentation is an ancient biotechnology to promote nutrient utilization and reduce antinutritional factors (ANFs) levels [40]. The probiotics prepared by SSF have been reported to be superior to those from submerged fermentation (SMF) in improving the growth performance and intestinal microbiota balance in broilers and weaned pigs [41,42], and the bacteria obtained from solid state fermentation exhibit better tolerance to various kinds of environment stress [43]. Our results showed that the survival rate of the cells from SSF was higher than that from LSF no matter whether they were prepared by spray drying or cold-air drying, suggesting an obvious bacterial product production advantages of SSF. However, our study also found that the cells from SSF had significantly lower tolerance to acid stress than the cells from LSF, thus leading to the reduction in live bacteria number, eventually weakening bacterial probiotic function. Therefore, we further explored the differences in the tolerance of the bacteria obtained by the two fermentation methods under acid stress.

### 3.2. Differences in Cell Morphology and Cell Membrane Fatty Acid Composition

**Figure 2:**
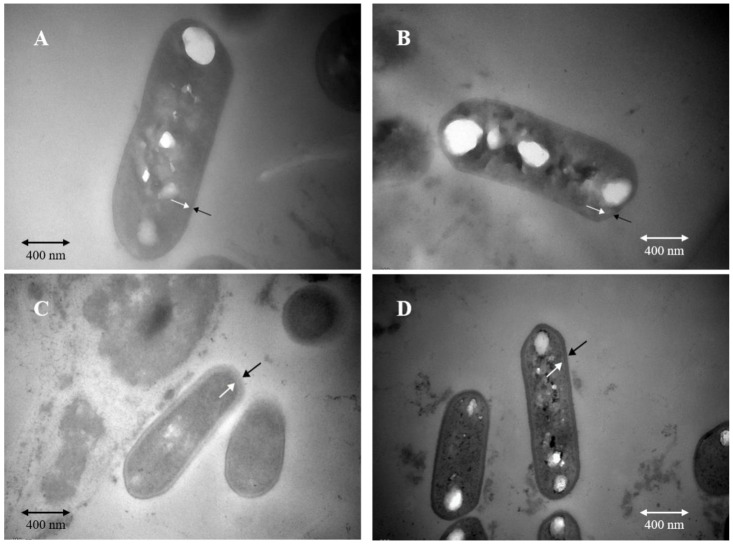
Transmission electron microscopy observation of cell morphological changes of *L. paracasei* Zhang under different stresses. (**A**). Cell morphology from solid-state fermentation before acid stress. (**B**) Cell morphology from liquid-state fermentation before acid stress. (**C**) Cell morphology from solid-state fermentation under pH 2 acid stress. (**D**) Cell morphology from liquid-state fermentation under pH 2 acid stress.

In order to explore morphological differences between the cells from two fermentations and cell morphological changes after acid stress treatment, we used transmission electron microscopy to observe the cell morphology. Before acid stress, the membrane structure of the cells from the two fermentation methods was intact, and the membrane wall was clear (Figure 2A,B). As shown in Figure 2C, after acid stress treatment, the membrane structure of cells obtained from SSF was damaged, and cell wall was blurred. By comparison, after acid stress treatment, the cell wall of the bacteria obtained from LSF was clear, and no cell membrane was damaged, which was not different from that before acid treatment (Figure 2D).

The cell membrane is a barrier between bacteria and the external environment, and it plays an important role in resisting the stimulation of the external environment and maintaining the stability of intracellular environment [44]. Since a large number of metabolic enzymes and signal receptors are distributed on the membrane, the cell membrane plays a very important role in the physiological metabolism of cells and signal energy conduction. Therefore, cell structure, cell membrane integrity, and cell membrane lipid composition are critical to the tolerance of the cells to environmental stimuli [45,46]. In this study, we found that the cell membrane obtained from liquid-state fermentation was more tolerant to acid stress, we speculated that it may be due to the fact that LSF was more beneficial to cell structure integrity maintenance.

Membrane fatty acid can maintain cellular viability under different conditions, and the relationship between membrane fatty acid composition and environmental stimuli is complex [47,48,49]. In LAB, temperature has been reported to induce changes in fatty acids such as the proportion of unsaturated fatty acids, the degree of cyclization, and the proportion of long-chain fatty acids containing 20 to 24 carbons [50,51]. Changing the cell membrane composition of fatty acids can effectively regulate the fluidity of the cell membrane and a response of cells to environmental stress [52,53]. In order to explore the differences in cell membrane fatty acid composition between the two types of cells obtained respectively by the two fermentation methods (SSF and LSF) under acid stress, we measured the membrane fatty acid of two types of cells. The results showed that (Table 1) the proportion of unsaturated fatty acids (palmitoleic acid C16:1, octapenoic acid C18:1ω 8C, oleic acid C18:1ω 9C, linoleic acid C18:2, linolenic acid C18:3, and docoenoic acid C22:1) in cell membrane of the bacteria obtained by solid state fermentation was significantly lower than that of saturated fatty acids (myristic acid C14:0, pentadecanoic acid C15:0, palmitic acid C16:0, heptadecanoic acid C17:0, stearic acid C18:0, and arachidic acid C20:0) with the ratio of saturated to unsaturated fatty acids (S/U) of 1.92, whereas the ratio of saturated fatty acids to unsaturated fatty acids (S/U) in the cell membrane from liquid state fermentation was 0.95, indicating the content of unsaturated fatty acids and saturated fatty acids in cell membrane was similar. Under acid stress, the content of saturated fatty acids in the cell membrane of the two types of cells increased, and the S/U ratio in solid-state fermented cells increased to 2.64, while the S/U ratio in liquid-state fermented cells increased to 3.92, thus the content of saturated fatty acids in liquid-state fermented cells increased more significantly than that in solid-state fermented cells.

**Figure 3:**
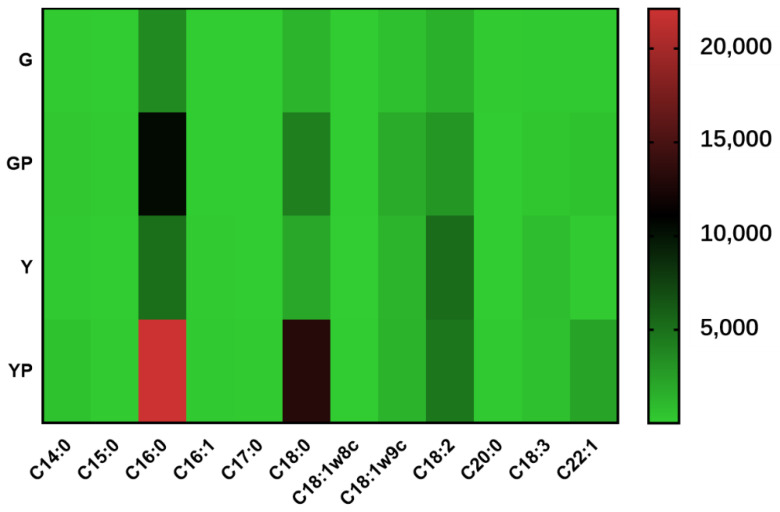
Heat map of fatty acid composition in cell membrane. G, solid state-fermented cells; Y, liquid state-fermented cells. GP, solid state-fermented cells treated with pH 2 for 10 min; YP, liquid state-fermented cells treated with pH 2 for 10 min. The numbers on the right side indicate the fatty acid content (μg/g). Results are based on three technical replicates.

Before acid stress treatment, palmitic acid, stearic acid, oleic acid, and linoleic acid exhibited high content in the cell membranes of the two types of cells (Figure 3). After acid stress treatment, in solid-state fermented cells, the content of linoleic acid in the cell membrane increased significantly from 1530.59 μg/g to 2972.23 μg/g, and the content of oleic acid also increased significantly from 682.13 μg/g to 1746.20 μg/g, However, under acid stress, in liquid-state fermented cells, the content of linoleic acid of the cells decreased from 5241.26 μg/g to 4685.70 μg/g; the content of oleic acid increased slightly from 1320.58 μg/g to 1351.64 μg/g; the content of palmitic acid in the cell membrane increased significantly from 5036.58 μg/g to 22,115.55 μg/g; while that of stearic acid increased significantly from 2025.53 μg/g to 13,065.27 μg/g. This might explain the reason for the significant changes in the S/U ratio of cell membrane fatty acids. The S/U ratio in the cells of *Lactobacillus plantarum* ZDY2013 (recently reassigned as *Lactiplantibacillus plantarum* ZDY2013) under acid stress was significantly higher than that without acid stress [26], which was consistent with our results that saturated fatty acid saturation in cell membranes of *Lacticaseibacillus paracei* Zhang increased under acid stress, thus improving the cell membrane rigidity in response to acid stress stimulation. The content of palmitic acid in *Lactobacillus casei* ATCC 334 (recently reassigned as *Lacticaseibacillus paracasei* ATCC334) increased under acid stress [54]. In contrast to the findings reported by Wu et al. that unsaturated fatty acid under acid stress increased [55], our results indicated that the S/U ratio in cell membranes increased after acid stress, and the increased content of two saturated fatty acids (palmitic acid and stearic acid) enhanced the rigidity of cell membrane, which was beneficial to tolerance of *Lacticaseibacillus paracei* Zhang to acid stress.

### 3.3. Differences in Intracellular Microenvironment and Activity of Metabolic Enzymes after Acid Stress Treatment

In order to reveal physiological differences of bacteria obtained by two fermentation methods under acid stress, we measured intracellular pH, intracellular ATP content, H^+^-ATPase and glutathione reductase activities of the bacteria.

**Figure 4:**
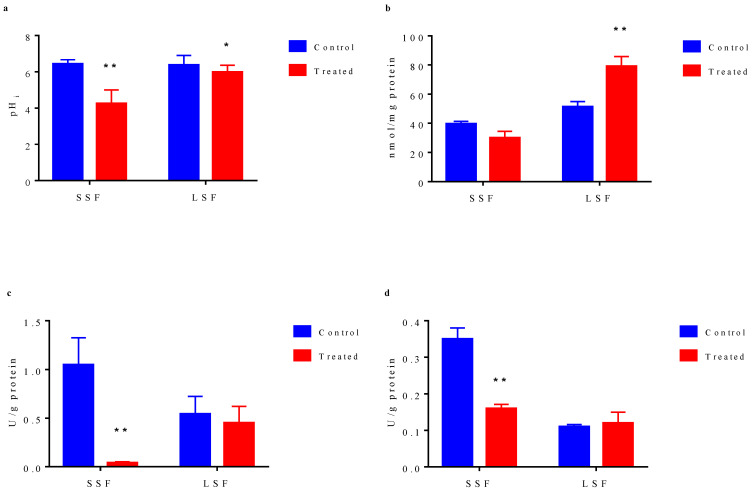
Physiological characteristic differences of cells cultured from solid-state fermentation and liquid-state fermentation. (**a**) Changes in intracellular pH. (**b**) Intracellular ATP concentration. (**c**) Activity of ATPase. (**d**) Activity of glutathione reductase. In bar chart, blue bar represents the cells without acid stress treatment, red bar indicates the cell exposed to pH 2.0 acid stress for 10 min. The results represent the mean of three duplicate experiments for each sample. Error bar indicates the mean ± standard deviation of three biological and three technical replicates. * statistically significant differences (*p* < 0.05) **, statistically significant differences (*p <* 0.01).

The results showed (Figure 4a) that before acid stress treatment, the intracellular pH values of the bacteria from the two fermentations were basically the same (6.43 for solid state and 6.38 for liquid state). After acid stress treatment, intracellular pH values of two types of bacteria were decreased. The intracellular pH of the liquid-state fermented bacteria decreased from 6.38 to 5.98, but basically maintained around 6.0. However, that of the solid-state fermented bacteria decreased significantly from 6.43 to 4.25, indicating that significant damage of the intracellular microenvironment by acid stress, which would seriously affect the activity of important intracellular metabolic enzymes and many important metabolic processes. The higher ability of liquid state-fermented bacteria to maintain intracellular pH under acid stress might be one of the reasons for their better tolerance to acid stress. Since the energy charge of the cell is an important indicator evaluating intracellular microenvironment, we measured the content of ATP in the cell. As shown in Figure 4b, before acid stress treatment, the content of ATP in cells from liquid-state fermentation (51.28 nmol/mg protein) was higher than that from solid-state fermentation (39.51 nmol/mg protein). After acid stress treatment, ATP content in solid-state fermented bacteria decreased to 30.08 nmol/mg protein, while ATP content in liquid-state fermented bacteria significantly increased to 79.15 nmol/mg protein. ATP and other energy substances can help cells to cope with the stimulation of adverse environment. The high ATP content in liquid state-fermented bacteria and the further increased intracellular ATP content induced by acid stress can maintain sufficient energy to cope with the adverse environment.

The change in intracellular microenvironment has most direct effect on the activity of various intracellular metabolic enzymes. Therefore, we measured the activities of intracellular H^+^-ATPase and glutathione reductase involved in environmental stress response. There are many kinds of ATPase in cells, of which H^+^-ATPase is a very important one, and it could maintain the pH balance in cells. Before acid stress treatment, H^+^-ATPase activity (1.05 U/g protein) of the bacteria from solid state fermentation was higher than that from liquid-state fermentation (0.54 U/g protein) (Figure 4c). After acid stress treatment, in solid-state fermented cells, H^+^-ATPase activity decreased significantly to 0.04 U/g protein, which was close to a complete activity loss, whereas in liquid-state fermented cells, H^+^-ATPase activity decreased slightly from 0.54 U/g protein to 0.45 U/g protein. Although H^+^-ATPase activity in solid-state fermented cells was higher than that in liquid-state fermented cell, H^+^-ATPase stability in solid-state fermented cells was obviously lower than that in liquid-state fermented cells under acid stress. Glutathione reductase (GR) can catalyze NADPH-dependent reduction of GSH disulfide. Similarly, our data indicated that although before acid stress, the GSH reductase activity in solid-state fermented cells (0.35 U/g protein) was higher than that in liquid-state fermented cells (0.11 U/g protein), the enzyme activity of solid-state fermentation bacteria significantly decreased to 0.16 U/g protein under acid stress, while the enzyme activity in the liquid-state fermented cells basically remained unchanged (0.12 U/g protein) (Figure 4d).

The stability of the intracellular environment, especially the stability of pH, is the prerequisite of the normal physiological responses of cells. If the intracellular pH cannot be maintained at a level close to neutral, the activity of important metabolic enzymes will be affected [56,57], resulting in the decreased cell activity or even cell death [58]. In this study, liquid-state fermented cells exhibited a better ability to regulate intracellular pH; thus, the activity of many important metabolic enzymes could be maintained better, including H^+^-ATPase and glutathione reductase, to ensure the normal intracellular physiological metabolism under acid stress. Some studies have shown that when confronted with acid stress conditions, LAB will resist acid stress by increasing the expression level of H^+^-ATPase or increasing the activity of H^+^-ATPase [59]. In addition, some studies have revealed that the expression of H^+^-ATPase is related to the response of *Lactobacillus casei* (recently reassigned as *Lacticaseibacillus paracasei*) to the acidic environment stress [60]. Our results demonstrated that liquid-state fermented cells had a better ability to maintain its H^+^-ATPase activity; thus, they could better regulate themselves when confronted with acid stress to maintain the stability of intracellular microenvironment. H^+^-ATPase needs the energy from ATP to function properly. The removal of protons from the cells requires the consumption of ATP, and some amino acid metabolic pathways produce ATP and ammonia, and the produced ammonia can further neutralize the protons in the cells to maintain the relative stability of intracellular pH [61]. *Lactococcus Lactis* NZ9000 has been reported to increase the intracellular ATP concentration in response to acid stress [62]. High H^+^-ATPase activity and sufficient energy guarantee the ability of cells to regulate intracellular pH homeostasis [63]. Our results also showed that the intracellular ATP content in liquid-state fermented cells increased significantly under acid stress stimulation; thus, the cells had higher energy maintenance ability, enabling H^+^-ATPase to function continuously, eventually leading to better response to acid stress. Glutathione reductase can maintain a high ratio of reduced glutathione to oxidized glutathione in cells, and it can act as an electron donor to assist cells to resist osmotic stress, toxic stress, and oxidative stress [64,65,66]. As an electron donor, glutathione can also scavenge reactive oxygen species [67]. This study revealed that liquid-state fermented bacteria had better ability to maintain the enzyme activity, which was conducive to bacterial tolerance to environmental stress. Our results of the intracellular microenvironment and metabolic enzyme activity assays were consistent with our analysis results of the difference in bacterial tolerance to acid stress. The higher tolerance to acid stress of the liquid-state fermented bacteria might be attributed to their better ability to maintain the stability of intracellular microenvironment and activity of metabolic enzymes.

### 3.4. Transcriptomic Differences between L. paracasei Zhang from SSF and LSF

**Figure 5:**
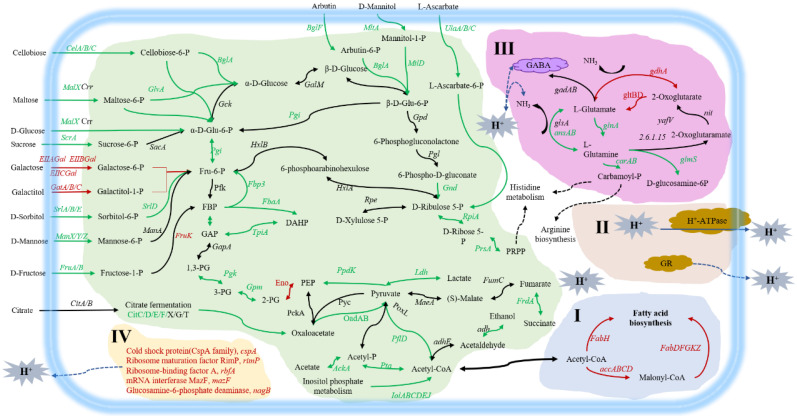
Schematic diagram of metabolism differences and acid stress tolerance mechanisms of *L.* *paracasei* Zhang obtained from LSF. The serial number (I, II, III, IV) indicates the acid stress tolerance mechanism of cells from LSF. (**I**) improving fatty acid metabolic pathway and changing the composition of cell membrane fatty acid; (**II**) maintaining the stability of intracellular microenvironment, maintaining and increasing enzyme activity; (**III**) changing glutamate metabolism, producing ammonia and GABA to alleviate acid stress; (**IV**) upregulating the expression of several genes related to acid stress response. Glu-6-P, glucose-6-phosphate; Fru-6-P, fructose-6-phosphate; FBP, fructose-1,6-diphosphate; DHAP, dihydroxyacetone-phosphate; GAP, glyceraldehyde-3-phosphate; 1,3-PG, 1,3-diphosphoglycerate; 3-PG, 3-phosphoglycerate; 2-PG, 2-phosphoglycerate; PEP, phosphoenolpyruvate; GR, Glutathione reductase. Red color indicates up-regulation; black color represents no change; green color indicates down-regulation at mRNA level. The blue lines show transportation, neutralization, and other coping processes.

We used the BGISeQ-500 platform to analyze the differences at the transcriptional level between solid-state fermented and liquid-fermented bacteria. The number of reads obtained from platform sequencing was shown in Table S1. The alignment between reads and reference genome (including genes) was shown in attached Table S2. Correlation analysis results of different replicates showed (Figure S3) that the correlation coefficients of samples from different replicates in the same group were all above 0.96, indicating a good correlation between different replicates in the same group, while the correlation coefficients among different groups were all below 0.56, indicating a low correlation between different groups. The results of principal component analysis also showed (Figure S4) that the three replicate samples in the same group were clustered together, and that they were obviously distinguishable from those in the different groups, indicating that there was an obvious analyzable difference between the groups. Transcriptional analysis revealed a total of 970 differentially expressed genes (DEGs, fold change > 2.0, *p* value < 0.001 LSF vs. SSF) (Figure S2), of which 687 DEGs were down-regulated and 283 DEGs were up-regulated. The GO and KEGG analyses of DEGs were performed, and the results were shown in Figures S5 and S6. The major metabolic differences were shown in Table S3 and Figure 5.

### 3.5. Expression Change of Genes Related to Phosphotransferase System (PTS)

Phosphotransferase system (PTS) is widely used in prokaryotes and archaea, through which prokaryotes transport carbohydrate or other substances to adapt to the environment and save resources. Specific transporter components in PTS system specifically deliver substances into the cells. Compared with the cells from solid-state fermentation, the cells from liquid-state fermentation exhibited an extensively reduced the expression of the genes related to sugar transport, especially those genes in the PTS system (Table S1).

In liquid-state fermented cells, the expressions of 9 genes related to mannose transport (including 4 *manX* genes, 2 *manY* genes, 2 *manZ* genes and 1 *manA* gene) were down-regulated by 2.01–39.63 times. These 9 genes were responsible for the transport of D-mannose from extracellular to intracellular to form mannose 6-phosphate, and the obtained mannose 6-phosphate was further catalyzed by mannose-6-phosphate isomerase encoded by gene *manA* to form fructose-6-phosphate.

The expressions of 10 genes (3 *celA* genes, 4 *celB* genes, and 3 *celC* genes) related to cellobiose transport were down-regulated by 2.02–9.02 times. These 10 genes were responsible for the transport of cellobiose from extracellular to intracellular to form cellobiose-phosphate. The resultant cellobiose-phosphate was further catalyzed by β-glucoside-6-phosphate isomerase to form α-D-glucose-6-phosphate. The expressions of three *bglA* genes encoding β-glucoside-6-phosphate isomerase were down-regulated by 3.01–38.85 times.

Five genes (2 *malX* genes, 1 *galU* gene, 1 *rfbB* gene, and 1 *pgi* gene) associated with the maltose/glucose transport, which were responsible for converting extracellular D-glucose and maltose to intracellular D-glucose 6-phosphate and maltose 6-phosphate, were down-regulated by 2.37 to 9.48 times. Meanwhile, the expression of gene *glvA* was down-regulated by 12.80 times, and this gene was responsible for further catalyzing the formation of D-glucose 6-phosphate and D-glucose from maltose 6-phosphate.

Six genes (2 *srlA* genes, 2 *srlB* genes, and 2 *srlE* genes) associated with the glucitol/sorbitol transport, which transformed extracellular D-sorbitol into intracellular sorbitol-6-phosphate, were down-regulated by 4.10-4.84 times. In addition, 2 *srlD* genes were down-regulated by 2.40 and 4.01 times, respectively, and these 2 genes further catalyzed sorbitol-6-phosphate into fructose-6-phosphate.

Four genes (2 *fruA* genes and 2 *fruB* genes) associated with the fructose transport, which converted extracellular D-fructose into intracellular fructosy-1-phosphate, were down-regulated by 2.15–3.0 times. The resultant fructosy-1-phosphate was further catalyzed into fructose-1,6-bisphosphatase (FBP) by the fructose-1-phosphate kinase encoded by intracellular gene *fruK*.

One gene (*scrA*) associated with sucrose transport, which converted extracellular sucrose into intracellular sucrose 6-phosphate, was down-regulated by 3.86 times. Another gene *sacA* encoding fructofuranosidase further catalyzed sucrose 6-phosphate to form α-D-glucose-6-phosphate and D-fructose. D-fructose further formed D-fructose-1-phosphate and FBP by double-phosphorylation.

Two *mtlA* genes involved in mannitol transport, which converted extracellular mannitol to intracellular mannitol-1-phosphate, were down-regulated by 2.43 times and 2.76 times, respectively. In addition, Gene *mtlD* encoding mannitol-1-phosphate 5-dehydrogenase was down-regulated by 2.75 times, which further catalyzed mannitol-1-phosphate into β-D-glucose6-phosphate.

Three genes (*UlaA*, *UlaB*, and *UlaC)* associated with ascorbate transport were down-regulated by 3.14–3.83 times, and these 3 genes converted extracellular L-ascorbate into intracellular L-ascorbate-6-phosphate. The obtained L-ascorbate-6-phosphate was further catalyzed to form xylulos-5-phosphate in the cell, and xylulos-5-phosphate participated in the pentose phosphate pathway.

Five genes (*citC, citD, citE, citF,* and *frdA*) encoding the citrate lyase in the two-component system were also down-regulated by 5.75–41.32 times, and the encoded citrate lyase catalyzed citric acid to form oxalacetic acid and acetic acid.

However, 10 genes (*lacA, lacD, lacB, galK, gatC, lacC, gatA, gatB, galE,* and *galT*) related to galactose and galactitol transport were up-regulated by 2.75–9.20 times. These 10 genes were responsible for transporting extracellular galactose and galactitol into the cells to form galactose-6-phosphate and galactitol-1-phosphate which were further converted into fructose-6-phosphate, and the obtained fructose-6-phosphate participated in the glycolysis and pentose phosphate pathways.

In general, compared with that in the cells from solid-state fermentation, the transport of various carbohydrates in the cells from liquid-state fermentation was weakened. The possible reason might lie in that the sugars were in a dissolved state in the liquid matrix; thus, the absorption, transport, and utilization of carbohydrates were easier. The expression upregulation of genes related to galactose transport in cells from liquid-state fermentation is worth further study.

### 3.6. Expression Change of Genes Related to Glycolysis, Pentose Phosphate Pathway, and Pyruvate Metabolism

Some genes related to glycolysis were down-regulated in the liquid-state fermented cells, compared with the solid-state fermented cells. Specifically, the expression of the gene *fbaA* encoding fructose-bisphosphate aldolase was down-regulated by 8.45 times, and the encoded fructose-bisphosphate aldolase catalyzed the reversible reaction between fructose-1,6-2-phosphate and dihydroxyacetone phosphate or glyceraldehyde triphosphate. Gene *fbp3* encoding fructose-1,6-bisphosphatase III was down-regulated by 2.64 times. *Gene tpiA* encoding the triphosphate isomerase was down-regulated by 3.80 times, and the encoded triphosphate isomerase catalyzed a reversible reaction between dihydroxyacetone phosphate and glyceraldehyde triphosphate. Gene *pgk* encoding phosphoglycerate kinase was down-regulated by 2.85 times, and the enzyme encoded by this gene catalyzed a reversible reaction between 1,3–2 phosphate and glycerate-3-phosphate. Two *gpmA* genes encoding 2,3-bisphosphoglycerate-dependent phosphoglycerate mutase were down-regulated by 2.07 times and 2.51 times, respectively, and the enzyme encoded by these two genes catalyzed a reversible reaction between glycerate-3-phosphate and glycerate-2-phosphate. However, gene *eno* encoding enolase was up-regulated in the liquid-state fermented cells. In general, the expression change of the gene related to glycolysis indicated the downstream glycolysis metabolism were partially weakened, and metabolic flow was changed.

Gene *gnd* encoding 6-phosphogluconate dehydrogenase was down-regulated by 9.45 times, and this enzyme catalyzed 6-phospho-D-gluconate into 5-phospho-D-ribulose and NADPH. The gene *rpiA* encoding ribose 5-phosphate isomerase A was down-regulated by 2.32 times, and this encoded enzyme catalyzed the formation of 5-phospho-D-ribose from 5-phospho-D-xylulose. Gene *prsA* encoding ribose-phosphate pyrophosphokinase was down-regulated by 2.85 times, and this encoded enzyme catalyzed 5-phospho-D-ribose into phosphate ribose pyrophosphate. Overall, the expressions of all the investigated genes related to the pentose phosphate pathway were down regulated, indicated this pathway was partially weakened in liquid-state fermented cells, compared with solid-state fermented cells.

Three genes (two *oadA* and one *oadB*) were down-regulated by 2.02 to 12.55 times. These three encoded the oxaloacetic decarboxylase subunit, and this enzyme subunit converted oxaloacetic acid into pyruvate in the cell. Gene *ldh* was down-regulated by 5.75 times. This gene encoded L-lactate dehydrogenase, and this encoded enzyme catalyzed pyruvate to produce lactate. Gene *pflD* encoding formate C-acetyltransferase was down-regulated by 2.86 times, and this encoded enzyme catalyzed the reversible conversion of pyruvate into acetyl CoA. In general, the metabolism of pyruvate into lactate and succinate was weakened in the liquid fermented cells. In addition, the gene *pta* encoding phosphate acetyl transferase was down-regulated by 3.89 times, and this encoded enzyme catalyzed the conversion between acetyl-CoA and acetyl-phosphate. Gene *ackA* encoding acetate kinase was down-regulated by 37.10 times, and this enzyme further catalyzed the conversion of acetyl-phosphate into acetate. Overall, the metabolic flow of pyruvate was changed.

### 3.7. Expression Change of Genes Related to Inositol Phosphate Metabolism and Fatty Acid Synthesis

Seven genes (*iolA, iolB, iolC, iolD, iolE, iolJ,* and *tpiA*) related to inositol phosphate metabolism in the cells obtained from liquid-state fermentation were down-regulated by 2.0–3.8 times, compared with the cells obtained from solid-state fermentation. These genes encoded the enzymes that converted inositol to acetyl-CoA and dihydroxyacetone-phosphate; thus, the inositol phosphate metabolic pathway in liquid-state fermented cells was weakened.

However, we found that genes *accA, accB, accC, and accD* encoding acetyl-CoA carboxylase were up-regulated by 6.42–10.04 times in the cells from liquid-state fermentation, and this enzyme encoded by these genes catalyzed acetyl-CoA into malonyl-CoA in the cells. In addition, seven genes associated with fatty acid synthesis (*fabF, fabG, fabD, fabK, fabH*, and 2 *fabZ*) were up-regulated by 6.66–14.60 times, resulting in the enhancement of fatty acid synthesis, which was opposite to the observation for *L. plantarum* (recently reassigned as *Lactiplantibacillus plantarum*) exposed to butanol (alcohol) stress [68] but in line with the previous findings that *Lactococcus lactis* could improve the expression of genes *acc* and *fab,* thus enhancing fatty acid synthesis under acid stress [21]. These results indicated that the fatty acid metabolism of lactic acid bacteria varied in response to different environmental stresses.

### 3.8. Expression Change of Genes Related to Glutamate Metabolism Pathways

Our data indicated that the pathways associated with glutamate metabolism were altered in the liquid-state fermented cells, compared with the solid-state fermented cells. In liquid-state fermented cells, the gene *glnA* encoding glutamine synthetase was down-regulated by 5.63 times, and the encoded enzyme catalyzed the conversion of glutamate into glutamine. Gene *ansAB* encoding L-asparaginase was down-regulated by 2.71 times, and this enzyme catalyzed the conversion of glutamate and glutamine. Two genes (*carA* and *carB*) encoding the small and large subunit of carbamyl-phosphate synthase were down-regulated by 5.31 and 6.15 times, respectively, and this enzyme catalyzed the conversion of glutamine to carbamyl-phosphate. Gene *glmS* encoding glucosamine-fructose-6-phosphate aminotransferase was down-regulated by 6.21 times, and the enzyme encoded by this gene catalyzed the conversion of L-glutamine into D-glucosamine-6-phosphate. Overall, the transition from glutamate to glutamine and many glutamate metabolism pathways were inhibited. However, genes *gltB* and *gltD* encoding the small and large chains of glutamate synthase (NADPH/NADH) were up-regulated by 12.06 and 12.16 times, and the encoded glutamate synthase catalyzed the conversion of 2-oxy-glutarate into glutamate, respectively. Gene *gdhA* encoding glutamate dehydrogenase was up-regulated by 3.62 times, and this glutamate dehydrogenase catalyzed the cross-conversion between 2-oxy-glutarate and glutamate, in which ammonia was released. Generally, glutamate was accumulated in the cells to produce γ-aminobutyric acid (GABA) under the catalysis of glutamate decarboxylase, thus relieving the acidic stress in the cell. This result was consistent with previous reports on GABA production as an important strategy for LAB to respond to acid stress [63,69].

### 3.9. Expression Change of Genes Related to Quorum-Sensing and Acid Stress Response

*CspA*, a gene encoding cold shock protein, was up-regulated by 7.29 times in the liquid-state fermented cells compared with the solid-state fermented cells. This gene has been reported to be involved in the response to cold stress and acid stress [70]. Gene *rimP* encoding ribosome maturation factor was up-regulated by 2.58 times. This gene in *Mycobacterium fortuitum* is involved in acid stress response [71]. Gene *rbfA* encoding ribosome-binding factor was up-regulated by 2.53 times, and this gene in *Lactobacillus plantarum* NMGL2 (recently reassigned as *Lactiplantibacillus plantarum* NMGL2) has been reported to be involved in the response to acid stress and cold stress [72]. Gene *mazF* was up-regulated by 2.25 times in liquid-state fermented cells, and this gene has been found to be activated under acid stress in *Bifidobacterium longum* [73]. Gene *nagB* encoding glucosamine-6-phosphate deaminase was up-regulated by 2.08 times, and this enzyme might be associated with acid stress response of *Lactobacillus casei* Zhang (recently reassigned as *Lacticaseibacillus paracasei* Zhang) [30]. Twelve genes related to quorum sensing (including biofilm formation, conjugation, aggregation, and other processes) were up-regulated by 2.3 to 8.84 times. These genes might be involved in the response of bacteria to acidic environment.

### 3.10. Mechanism of Liquid-State Fermented Cells in Response to Acid Stress

Combining the analyses of acid stress tolerance at physiological and transcriptional levels, we summarized the mechanisms of liquid-state fermented bacteria in response to acid stress as (Figure 5): (I) Improving the fatty acid metabolism pathway and changing the composition of cell membrane fatty acid; (II) maintaining the stability of the intracellular microenvironment, maintaining and increasing enzyme activity; (III) changing the glutamate metabolism, producing ammonia and GABA to alleviate the acid environment; (IV) up-regulating the expression of several genes related to acid stress response. By these mechanisms, the cells from liquid-state fermentation made a good response to acid stress.

## 4. Conclusions

In this study, the biomass, survival rate of the bacterial agent, and the acid stress tolerance of the cells obtained from solid-state fermentation and liquid-state fermentation were investigated. The results indicated that the survival rate of the bacterial agent from SSF was higher than that from LSF. However, the bacteria from LSF were more tolerant to acid stress. Then, the differences at the physiological and transcriptomic levels were analyzed under acid stress. The data showed that for the liquid-state fermented cells, the stability of intracellular pH was higher, the activity of intracellular metabolic enzymes was lower but more stable, and the membrane structure and fatty acid composition were more conducive to bacterial adaptation to the acid environment. In addition, we found that genes related to the sugar transport, glycolysis, and the pentose phosphate pathway, inositol phosphate metabolism, and pyruvate metabolism were down-regulated in liquid-state fermented cells, and that the genes related to fatty acid synthesis, glutamate metabolism, and acid stress response were up-regulated. The gene expression regulation might be an important strategy for bacteria to respond to acid stress. In combination with physiological and transcriptional analyses, we summarized the mechanisms of liquid-state fermented bacteria in response to acid stress tolerance as the changes in cell membrane structures, the maintenance of intracellular stability and metabolic enzyme activity, and the expression change of genes related to acid stress response. Our study provides a novel insight into the mechanisms of acid stress response and offers a reference for the selection of LAB production methods.

## Figures and Tables

**Table 1:** Fatty acid composition of cell membrane under environmental stress.

FA or Parameter	Fatty Acid Distribution (μg/g, M/M %)			
	G	GP	Y	YP
C_14:0_	116.56 ± 12.69	309.84 ± 27.05	194.78 ± 10.13	595.60 ± 87.63
C_15:0_	36.93 ± 4.35	99.61 ± 8.87	29.30 ± 1.80	83.66 ± 7.74
C_16:0_	3589.90 ± 351.80	10,490.40 ± 773.05	5036.58 ± 220.69	22,115.55 ± 2961.51
C_16:1_	40.77 ± 5.92	66.61 ± 7.39	163.85 ± 9.60	230.47 ± 33.66
C_17:0_	24.45 ± 2.78	43.44 ± 3.61	29.22 ± 1.90	87.81 ± 8.62
C_18:0_	1341.62 ± 137.26	4190.22 ± 308.72	2025.53 ± 89.15	13065.27 ± 1781.62
C_18:1ω8c_	26.95 ± 4.79	26.84 ± 1.30	17.50 ± 1.00	34.00 ± 13.05
C_18:1ω9c_	682.13 ± 65.14	1746.20 ± 137.60	1320.58 ± 40.83	1351.64 ± 162.82
C_18:2_	1530.59 ± 163.38	2972.23 ± 221.16	5241.26 ± 214.60	4685.70 ± 543.25
C_18:3_	229.96 ± 23.94	366.59 ± 30.10	835.87 ± 36.46	686.66 ± 89.92
C_20:0_	140.07 ± 15.32	62.10 ± 4.66	35.95 ± 1.80	186.65 ± 16.90
C_22:1_	219.28 ± 25.63	588.33 ± 49.55	173.98 ± 6.75	2229.79 ± 311.61
Saturated/unsaturated ratio	1.92	2.64	0.95	3.92

Notes: G, solid state-fermented cells; Y, liquid state-fermented cells. GP, solid state-fermented cells treated with pH 2 for 10 min; YP, liquid state-fermented cells treated with pH 2 for 10 min. Results are based on three technical replicates.

## Data Availability

Data is contained within the article or supplementary material.

## Supplementary Materials

The following are available online at https://www.mdpi.com/article/10.3390/microorganisms9091951/s1, Figure S1: Survival rates of *L. paracasei* Zhang after spray drying and cold-air drying. ** statistically significant differences determined by Student’s t-test (*p <* 0.01). Figure S2: Volcano map of differentially expressed genes in the bacteria obtained by solid state fermentation and liquid state fermentation. Every point represents one gene. The blue points represent significantly down-regulated genes and red points represent significantly up-regulated genes, Figure S3: Pearson correlation between samples, Figure S4: Principal component analysis (PCA) between samples, Figure S5: GO enrichment analysis of DEGs in *L. paracasei* Zhang (SSF vs. LSF), Figure S6: KEGG pathway analysis of DEGs in *L. paracasei* Zhang (SSF vs. LSF), Table S1: Number of reads before and after trimming, Table S2: Information of read alignment against the reference genome and genes, Table S3: Genes differentially expressed at the transcriptional level in *L. paracasei* Zhang cultured from solid-state fermentation and liquid-state fermentation.
