# Supplementary material for: Differences in Acid Stress Response of Lacticaseibacillus paracasei Zhang Cultured from Solid-State Fermentation and Liquid-State Fermentation
Source: Microorganisms. 2021 Sep 14;9(9):1951. doi: 10.3390/microorganisms9091951 (PMC8465097; doi:10.3390/microorganisms9091951)
Supplement: Supplementary file 1 [file microorganisms-09-01951-s001.zip › microorganisms-1374035-supplementary - proofread-done.pdf]

microorganisms

Supplementary Material

Table S1. Number of reads before and after trimming.

Samples	Total raw reads (M)	Total clean reads (M)	Total clean bases (Gb)
SSF-1	21.93	21.45	1.07
SSF-2	21.93	21.50	1.07
SSF-3	21.94	21.56	1.08
LSF-1	21.94	21.01	1.05
LSF-2	21.84	21.10	1.05
LSF-3	21.94	21.04	1.05

Table S2. Information of read alignment against the reference genome and genes.

Samples	Alignment against reference genome			Alignment against reference genes		
	Total clean reads (M)	Total mapping (%)	Uniquely mapping (%)	Total clean reads (M)	Total mapping (%)	Uniquely mapping (%)
SSF-1	21.45	99.19	96.77	21.45	75.95	75.57
SSF-2	21.50	99.23	97.16	21.50	76.05	75.75
SSF-3	21.56	99.35	97.80	21.56	76.63	76.28
LSF-1	21.01	97.17	95.78	21.01	78.67	78.24
LSF-2	21.10	98.09	96.56	21.10	78.45	77.99
LSF-3	21.04	98.60	97.33	21.04	82.43	82.00

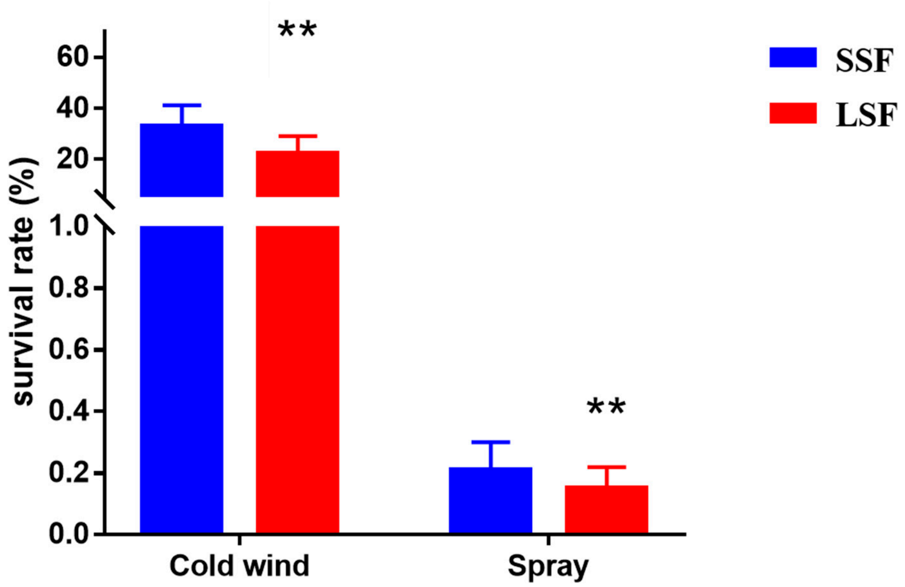

Figure S1. Survival rates of *L. paracasei* Zhang after spray drying and cold-air drying. **, statistically significant differences determined by Student's *t*-test ($P < 0.01$).

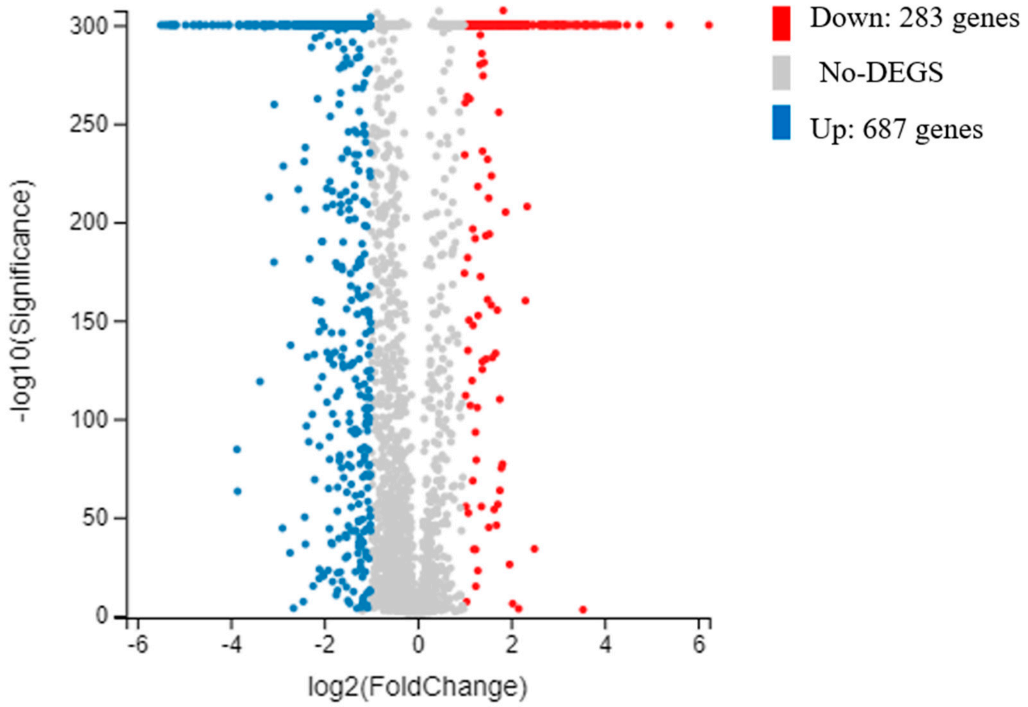

Figure S2. Volcano map of differentially expressed genes in the bacteria obtained by solid state fermentation and liquid state fermentation. Every point represents one gene. The blue points represent significantly down-regulated genes and red points represent significantly up-regulated genes.

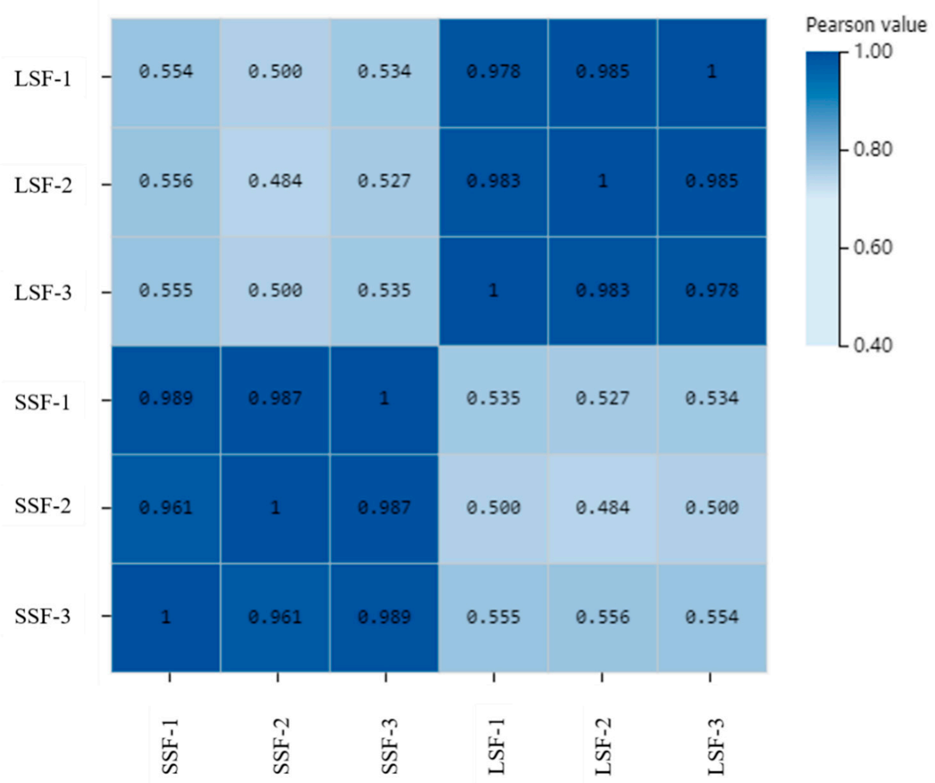

	SSF-1	SSF-2	SSF-3	LSF-1	LSF-2	LSF-3
LSF-1	0.554	0.500	0.534	0.978	0.985	1
LSF-2	0.556	0.484	0.527	0.983	1	0.985
LSF-3	0.555	0.500	0.535	1	0.983	0.978
SSF-1	0.989	0.987	1	0.535	0.527	0.534
SSF-2	0.961	1	0.987	0.500	0.484	0.500
SSF-3	1	0.961	0.989	0.555	0.556	0.554

Figure S3. Pearson correlation between samples.

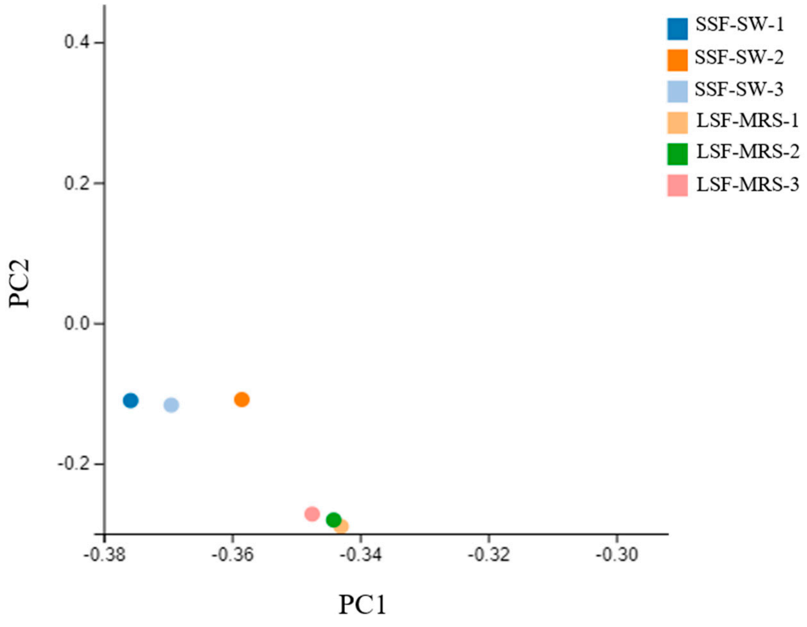

Figure S4. Principal component analysis (PCA) between samples.

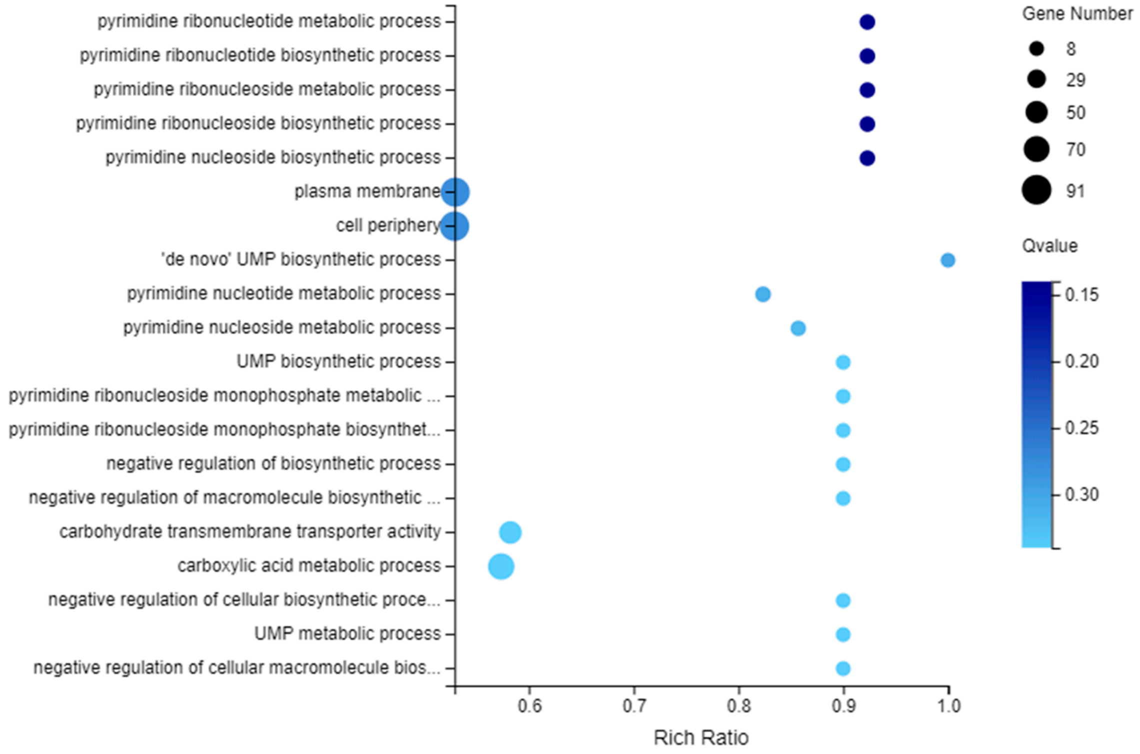

Figure S5. GO enrichment analysis of DEGs in *L. paracasei* Zhang (SSF Vs LSF).

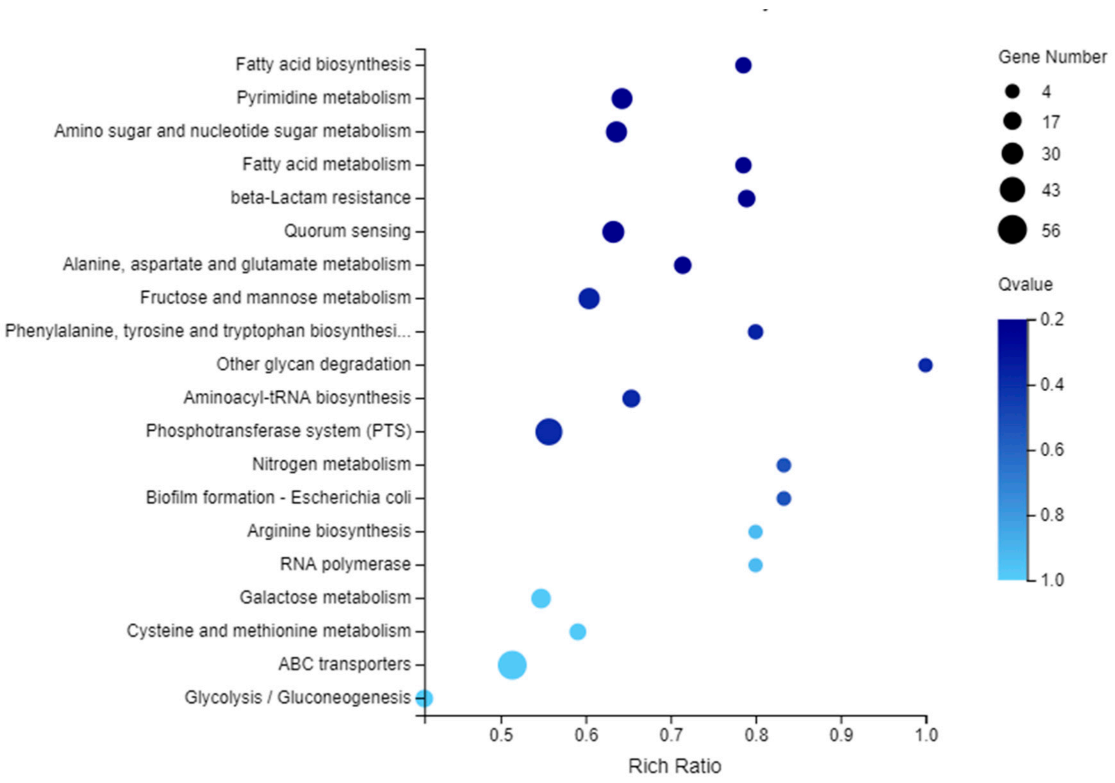

Figure S6. KEGG pathway analysis of DEGs in *L. paracasei* Zhang (SSF Vs LSF).
